# Maternal Common Mental Disorder as Predictors of Stunting among Children Aged 6-59 Months in Western Ethiopia: A Case-Control Study

**DOI:** 10.1155/2019/4716482

**Published:** 2019-03-10

**Authors:** Shimelis Girma, Teshale Fikadu, Eba Abdisa

**Affiliations:** ^1^Department of Psychiatry, Institute of Health, Jimma University, Ethiopia; ^2^Department of Epidemiology, Arbaminch University, Ethiopia; ^3^School of Nursing, Wollega University, Ethiopia

## Abstract

**Background:**

Child malnutrition in low- and middle-income countries still continues to be an alarming. Africa and Asia bear the greatest share of all forms of malnutrition. The association between maternal common mental disorder and stunting has not been studied well even in developed countries; much less in developing countries and even the findings are conflicting. Thus, the purpose of the present research was to investigate the relationship of maternal common mental disorder and child stunting.

**Methods:**

Institution based unmatched case-control study design was employed from March to April 2017. Two hundred thirty-four sampled children (78 cases and 156 controls) were randomly selected. Anthropometric measurements (height/length and weight) were taken by calibrated instruments. Maternal common mental disorder (CMD) was measured by using the locally validated Self-Reporting Questionnaire (SRQ-20). Data entry was done by Epi data version 3.1 and analysis was done by SPSS 21.0 statistical software.

**Result:**

Finding of this study found out about three-fourths of cases (71.8%) and three-fourths of controls (69.9%) were residing in rural and urban areas, respectively. Regarding maternal common mental disorder, more than half of cases mother (53.8%) and more than one-tenth of controls mother (13.5%) were found to have common mental disorder. The study showed that children of mothers who had common mental disorder were found to be three times more likelihood of developing stunting than children whose mothers had not common mental disorder.

**Conclusion and Recommendation:**

The study indicated that maternal common mental disorder was significantly associated with stunting. Therefore, emphasis should be given in preventing, managing, and maintaining maternal mental health in order to prevent stunting.

## 1. Background

Children morbidity and mortality secondary to malnutrition remain the major public health concern worldwide [1]. Every year 10 million children die from preventable illness and more than half is accountable to malnutrition [2]. Infant undernutrition is a well-recognized public health problem in low- and middle-income countries (LAMIC) [3–5], the cause of which extends beyond mere shortage of food [3, 6, 7]. There are several factors implicated as cause of malnutrition and one of it and the neglected part is maternal mental disorder.

Maternal CMD which is comprised of depressive, anxiety, and somatic symptoms is highly prevalent and it is one of the major contributors to the global burden of disease in low- and middle-income countries (LAMIC) [7–10]. Evidences of epidemiological studies had revealed that antenatal mental distress is a public health concern in sub-Saharan Africa [11, 12] and it is one of a risk factor that prevent children from attaining their growth potentials [8]. It has been associated with low infant birth weight, impaired postnatal growth, and increased frequency of infant diarrhoea [13], undernutrition, stunted growth, and poorer cognitive development [14].

The adverse role of maternal mental health on child nutrition and physical growth is increasingly recognized in LAMICs [15–18]. Factors that affect maternal mental health can adversely affect growth and development of infants. Maternal depressive symptom is risk factors for child physical growth and developmental problems; this can be related to child's susceptibility to common childhood illness particularly diarrheal diseases and child's fewer tendencies to be vaccinated [19, 20].

There are several factors implicated for the association of maternal CMD and child physical growth. One of the reasons for the association is poor maternal health seeking behaviour for childhood illness. Study found out that depressed mothers display poor health care seeking behaviours for their child's illness [21]. Maternal health can impact child feeding practice through different mechanisms. The interaction between mother and child is essential for caring the child and to effectively feed the child, while in the case of mental distressed mothers, the mothers can be more withdrawn in their interactions with their children and provide care for their children in different ways [21, 22]. Aside of this, the illness nature forces mother to have less demanding care for the child which further affects child's health care seeking behaviours and feeding practice [23] and also likely to interfere with the emotional quality of child care, which is a known risk factor for decreased nutrient intake, poor growth, and development [24, 25]. In line with this, good child care practices have been shown to be an effective means by which to mitigate the effects of poverty on children's nutrition [26].

Evidence regarding the link between maternal CMD and infant physical growth is conflicting. Studies from South Asia consistently reported that maternal depression was associated with impaired infant physical growth. Finding of studies indicated maternal depression was associated with stunting in India [1, 27, 28], Pakistan [28], Vietnam [27], and Bangladesh [29]. However, studies in Africa reported mixed findings. Thus, the present research was designed to address the limited knowledge and to investigate the clear relationship between child stunting and maternal common mental disorder.

## 2. Methods

### 2.1. Study Design and Subject

Community based unmatched case-control study design was employed. The study was conducted in Nekemte town which is located in East Wollega, Oromia regional state, Ethiopia. The town is situated in West of Ethiopia and covers a total area of 4,623 hectares. The town has a latitude and longitude of 7°40′N 36°50′E. The daily mean temperature of the town ranges from 20°c to 25°c year round and an average annual rain fall is 1500mm. In East Wollega zone, cereals contributed 88.9% of the grain crop area and 93.08% of the production, pulses cover 8.35% of the grain crop area, and red peppers and Ethiopian cabbage cover 47.1% and 44.6% of the area under vegetables, respectively. Administratively the town is divided into 13 Kebeles. Thus, the study was conducted in selected Kebeles of the town from March to April 2017.

### 2.2. Measurements

Data was collected from all eligible children and corresponding mothers by using semistructured and pretested interviewer administered questionnaires. Anthropometric data was collected using anthropometric measurement tools (MUAC tape, stadiometer, digital weight scale, and salter scale) from all children. Measurements were taken twice by different measurers for each child. Weight was measured using digital electronic measuring scale (SECA) to the nearest 0.1 kg on bare foot and with the minimum possible light clothes. Height/length was measured using standard procedure (Frankfurt position) in standing position using height measuring board/stadiometer to the nearest 0.1 cm for children who were 24 months and older. For children below 24 months of age, length was measured in a recumbent position. MUAC was measured using easily portable measurement device on left hand half way between the olecranon and acromion process by using armband/tape. Children were also assessed for the presence or absence of oedema of the feet. Maternal CMD was measured by using the locally validated Self-Reporting Questionnaire (SRQ-20). The questionnaire has twenty yes/no items asking about the experience of depressive, anxiety, panic, and somatic symptoms in the preceding 30 days. The total score was dichotomized with cut off points 6 (SRQ-20 < 6 and SRQ ≥ 6) in the current study.

Seven nurses were recruited as data collector; five nurses with their assistance were recruited as measurer and four nurses were recruited as a supervisor. Training was given for data collectors, measurers, and supervisors for three days on overall procedure of the study.

### 2.3. Participants

Source population for cases were all children aged 24–59 months in the district and who were stunted (height-for-age < -2 SD of median value of the WHO Child Growth Standards); all children aged 24–59 months in the district and who were not stunted (height-for-age ≥ -2 SD of median value of the WHO Child Growth Standards) were source population for cases and controls, respectively.

Sample size was computed by using STATCALC application of Epi Info 7 statistical software with the assumptions of proportion of households having family size of five and above among controls were 46.9 %; proportion of households having family size of five and above among cases were 68.14 %; 95 % confidence level, 80 % power of the study, case to control ratio of 1:2 to detect an odds ratio of 2.42 and adding 10 % of nonresponse rate. Thus, the sample size required for the study was 234 (78 cases and 156 controls). All children who fulfilled eligibility criteria in selected Kebeles were measured for their z-score of height-for-age and those who were stunted taken as sampling frame and the sample were allocated proportionally to the selected Kebeles based on number of stunted children. Simple random sampling method was used to recruit cases and the neighbouring nonstunted children were taken as control.

### 2.4. Statistical Analysis

Data was checked for completeness, edited, coded, and entered into Epi data version 3.1 and exported to SPSS version 21.0 statistical software for analysis. Descriptive statistics such as mean, median, frequency, and percentage was presented using charts and tables. Bivariate analysis was done and all explanatory variables which had association with the outcome variable with P-value less than 0.25 were included in multivariable analysis. Multivariable logistic regression analysis was employed to determine independent determinant factor among explanatory variables. Adjusted odds ratio (AOR), 95% confidence interval, and P-value less than or equal to 0.05 were used to decide statistically significant association with outcome variable. Anthropometric measurements were taken twice and a difference of 0.1 kg in weight and 0.1 cm in length/height was accepted as normal. However, mean results of repeated measurers were used upon significantly larger difference. After then, HAZ- scores were generated by WHO Anthro statistical software version 3.2.2. Wealth index was determined based on ownership of fixed assets and ownership of each fixed asset was given a value one and nonownership a value of zero. After checking for assumption, factor score was generated through principal component analysis and rank ordered in to five quintiles. Model fitness was assessed using Hosmer and Lemeshow test and it was found to be 0.75. Multicollinearity was checked by variance inflation factor (VIF) and tolerance test. The result of VIF was found to be less than 2 while tolerance test was greater than 0.1, which was within the normal limit.

## 3. Result

### 3.1. Sociodemographic Characteristics of Respondents

Two hundred thirty-four (78 cases and 156 controls) participants were included in the study. The mean age of the study participants was 17.2 ± 10.2 months (18.29 ± 10.23 months for cases and 16.6 ± 10.2 months for controls). The mean weight for cases was 6.6 ± 1.7 kg and 9.0± 2.9 kg for controls. The median height for cases was 85 ± 5.8 cm and for controls was 96 ± 7 cm. Almost half (53.7%) of cases and as well as controls were male with median birth orders for cases were 4 and for controls were 3 (Table 1).

### 3.2. Maternal CMD

In the current study, it was found out that 53.8% of cases mother and 13.5% of controls mother were found to have common mental disorder.

### 3.3. Other Factors

Finding of recent study found out thirty-four (43.6%) of cases and significant number of controls (12.8%) had at least one diarrhoea episode last two weeks prior of data collection period. Similarly, about two-thirds of study participant children (both cases and controls) were exclusively breastfed for six months (Table 2).

### 3.4. Multivariable Analysis

Multivariable logistic regression analysis indicated that children whose mothers having CMD were found to be three times more likelihood to be stunted than children whose mothers had no CMD (AOR=3.24, 95% CI, 1.14 to 9.21). Aside of this, the final model found out that children whose family were in lowest wealth index (AOR=3.76, 95% CI, 1.24 to 11.38), lack of maternal formal education (AOR= 4.08, 95% CI, 1.46 to 11.40), liquid waste disposal other than waste pit (AOR=5.95, 95% CI, 1.83 to 16.97), less frequent maternal hand washing practice (AOR=5.57, 95% CI, 1.82 to 16.97), diarrhoea report (AOR=3.58, 95% CI, 1.15 to 11.07), bottle feeding (AOR=3.98, 95% CI, 1.29 to 12.36), and poor maternal knowledge about child feeding (AOR=5.97, 95% CI, 1.83 to 19.44) were positively associated with stunting (Table 3).

## 4. Discussion

Stunting is a major public health problem in developing countries and the current prevalence in Ethiopia is 38.4% [30]. Different intervention strategy was used to reduce nutritional problem in the country but until recent time stunting prevalence is high and continues to become the major public health problem. There were several studies done to assess the determinant factors of stunting; however, those studies were failed to address the relationship between maternal common mental disorder and stunting. Thus, this study was designed to assess clear relationship between maternal CMD and stunting among children of age 6-59 months.

The current study found out children whose mothers had CMD were found to be three times more likelihood of stunting than children whose mothers had no CMD. The possible reason for this can be that maternal depression has an impact on child parenting related to poor interpersonal behaviour due to reduced maternal interest in a child [31] which further interfered with the emotional quality of child care [25] and reduced ability to provide a healthful diet for the child [32] which was supported by the finding of the same study in which case those children who fed complementary food using bottle were 3.98 times more likely hood to develop stunting than children who fed using cup or spoon. Study finding indicated that feeding child using bottle and hand were associated with increased odds of stunting [33, 34].

The illness nature of common mental disorder in mothers reduces their involvement in child care which impairs physical growth of the child [28] and also made mothers less responsive towards their children [35] and become less likely to form a secure attachment with their children [36]. Maternal mental disability, lack of family support, and lack of financial empowerment could impact infant care [37, 38]. Furthermore, research evidences indicated that depressive symptoms in mothers were associated with household food insecurity [39]. The association between mental health specifically depression and food insecurity is complex. A bidirectional causal relationship between household food insecurity and depression is documented [40] and different mechanisms such as psychological distress and micronutrient deficiency are reported [41, 42]

Child who had diarrhoea attack in the last two weeks prior to the survey was found to be about 4 times more likelihood for stunting than child who had no diarrhoea. In line with the recent finding, study results suggest that children of mothers with depressive symptoms are less likely to receive all follow-up vaccinations and have higher incidence of diarrheal disease and febrile illnesses which make them more susceptible to undernutrition [43, 44]. This can be due to excessive loss of fluids and electrolytes, loss of appetite, and lack of absorption of food during diarrhoea episodes. Similarly the study found out that children of mothers who less frequently wash their hands during critical period of child care were 5.57 times more likelihood of having stunting than children of mothers who frequently wash their hands. This can be due to the fact that deficit in maternal care such as poor hand washing practice, unsafe food preparation, and storage lead to more diarrheal episode of child of depressed mothers which further influence the nutritional status of children [45].

Furthermore, the study found out that child from family of lowest wealth index (AOR=3.76, 95% CI, 1.24 to 11.38), lack of maternal formal education (AOR= 4.08, 95% CI, 1.46 to 11.40), liquid waste disposal other than waste pit (AOR=5.95, 95% CI, 1.83 to 16.97), and poor maternal knowledge about child feeding (AOR=5.97, 95% CI, 1.83 to 19.44) were positively associated with stunting.

The main strength of the study was that the researchers use measurers other than data collectors in order to minimize interviewer bias and the design used was capable of evaluating the association of stunting to other exposure variables. The limitation of the study was that tools used to measure some explanatory variable were relied on self-report which was prone to recall and social desirability bias.

## 5. Conclusion and Recommendation

Community based case-control study from western rural Ethiopia found out that there is significant association between maternal common mental disorder and child stunting. This result further supports the link between maternal CMD and stunting in LAMICs. Accordingly, the researchers recommend scaling up maternal mental health care services and attention need to be given on integrating maternal mental health in existing maternal health routine cares.

## Figures and Tables

**Table 1:** Background characteristics of study population of Nekemte town, West Ethiopia, 2017.

Variable	Case (n = 78)	Control (n=156)	P-value
	N (%)	N (%)	
*Maternal CMD*			< 0.001
Yes	42(53.8)	21(13.5)	
No	36(46.2)	135(86.5)	
*Child sex*			< 0.001
Female	53(67.9)	57(36.5)	
Male	25(32.1)	99(63.5)	
*Place of residence*			< 0.001
Rural	56(71.8)	47(30.1)	
Urban	22(28.2)	109(69.9)	
*Birth order*			< 0.001
> 3	53(67.9)	43(27.6)	
1-3	25(32.1)	113(72.4)	
*Maternal age*			< 0.001
15-24	6(5.0)	9(7.4)	
25-34	52(43.0)	43(35.5)	
35-44	31(25.6)	37(30.6)	
*Maternal education*			< 0.001
No Formal Education	54(69.2)	48(30.8)	
Formal Education	24(30.8)	108(69.2)	
*Parental Marital status*			0.002
Divorced/Widowed	13(16.7)	7(4.5)	
Married	65(83.3)	149(95.5)	
*Wealth index*			< 0.001
Lowest	45(57.7)	29(18.6)	
Middle	5(6.4)	26(16.7)	
Highest	28(35.9)	101(64.7)	
*HFIAS Status*			< 0.001
Food Insecure	57(73.1)	57(36.5)	
Food Secure	21(26.9)	99(63.5)	
*Solid waste disposal site*			< 0.001
Other than waste pit	39(50.0)	37(23.7)	
Solid waste pit	39(50.0)	119(76.3)	
*Liquid waste disposal site*			< 0.001
Other than waste pit	48(61.5)	39(25.0)	
Liquid waste pit	30(38.5)	117(75.0)	
*Mother hand washing practice*			< 0.001
Less frequently	48(61.5)	32(20.5)	
More frequently	30(38.5)	124(79.5)	

**Table 2:** Maternal and child health care related factors of study participant of Nekemte town, West Ethiopia, 2017.

Variable	Case (n = 78)	Control (n=156)	P-value
	N (%)	N (%)	
*Place of delivery*			0.249
Home	15(19.2)	21(13.5)	
Health institution	63(80.8)	135(86.5)	
*ANC*			0.628
No	8(10.3)	13(8.3)	
Yes	70(89.7)	143(91.7)	
*History of diarrhoea*			< 0.001
Yes	34(43.6%)	20(12.8%)	
No	44(56.4%)	136(87.2%)	
*History of febrile illness*			0.284
Yes	23(29.5)	57(36.5)	
No	55(70.5)	99(63.5)	
*Vaccination status*			0.148
Incomplete	18(23.1)	24(15.4)	
Completed	60(76.9)	132(84.6)	
*Breast feeding initiation*			0.269
> 1 hour	21(26.9)	32(20.5)	
≤ 1 hour	57(73.1)	124(79.5)	
*Per lacteal feed*			0.114
Yes	18(23.1)	23(14.7)	
No	60(76.9)	133(85.3)	
*Colostrum*			0.571
Discarded	4(5.1)	11(7.1)	
Given to child	74(94.9)	145(92.9)	
*Duration of EBF*			0.900
< or > than 6 months	22(28.2)	42(26.9)	
For 6 months	56(71.8)	114(73.1)	
*DDS*			0.001
Low DDS	71(91.0)	111(71.2)	
High DDS	7(9.0)	45(28.8)	
*Bottle feeding*			< 0.001
Yes	37(47.4)	35(22.4)	
No	41(52.6)	121(77.6)	
*Way of feeding*			0.714
All children together	6(7.7)	10(6.4)	
With separate dish	72(92.3)	146(93.6)	
*Maternal Knowledge*			<0.001
Poor	45(57.7)	48(30.8)	
Fair	18(23.1)	21(13.5)	
Good	15(19.2)	87(55.8)	

**Table 3:** Independent predictors of stunting of study participants of Nekemte town, West Ethiopia, 2017.

Variable	Case (n = 78)	Control (n=156)	COR(95% CI)	AOR(95% CI)
	N (%)	N (%)		
*Maternal CMD*				
Yes	42(53.8)	21(13.5)	*7.5(3.96, 14.22)* ^**∗∗∗**^	*3.24(1.14,9.21)* **** ^**∗**^
No	36(46.2)	135(86.5)	1	1
*Child sex*				
Female	53(67.9)	57(36.5)	*3.68(2.07,6.55)* **** ^**∗∗∗**^	2.99(0.07,8.38)
Male	25(32.1)	99(63.5)	1	1
*Wealth index*				
Lowest	45(57.7)	29(18.6)	*5.59(2.99,10.48)* **** ^**∗∗∗**^	*3.76(1.24,11.38)* **** ^**∗**^
Middle	5(6.4)	26(16.7)	0.69(0.24,1.97)	0.49(0.09,2.52)
Highest	28(35.9)	101(64.7)	1	1
*Birth order*				
above 3	53(67.9)	43(27.6)	*5.57(3.09,10.06)* **** ^**∗∗∗**^	4.25(0.44,12.53)
1-3	25(32.1)	113(72.4)	1	1
*Maternal education*				
No Formal Education	54(69.2)	48(30.8)	*5.06(2.81,9.12)* **** ^**∗∗∗**^	*4.08(1.46,11.40)* **** ^**∗∗**^
Formal Education	24(30.8)	108(69.2)	1	1
*Liquid waste disposal site*				
Other than waste pit	48(61.5)	39(25.0)	*4.8(2.68, 8.59)* **** ^**∗∗∗**^	*5.95(1.83,16.97)* **** ^**∗∗**^
Liquid waste pit	30(38.5)	117(75.0)	1	1
*Mother hand washing*				
Less frequently	48(61.5)	32(20.5)	*6.2(3.41, 11.29)* **** ^**∗∗∗**^	*5.57(1.82,16.97)* **** ^**∗∗**^
Frequently	30(38.5)	124(79.5)	1	1
*History of Diarrhoea*				
Yes	34(43.6%)	20(12.8%)	*5.25(2.75,10.05)* **** ^**∗∗∗**^	*3.58(1.15,11.07)* **** ^**∗**^
No	44(56.4%)	136(87.2%)	1	1
*Bottle feeding*				
Yes	37(47.4)	35(22.4)	*3.12(1.74, 5.59)* **** ^**∗∗∗**^	*3.98(1.29,12.36)* **** ^**∗**^
No	41(52.6)	121(77.6)	1	1
*Maternal Knowledge*				
Poor	45(57.7)	48(30.8)	*5.44(2.75,10.76)* **** ^**∗∗∗**^	*5.97(1.83,19.44)* **** ^**∗∗**^
Fair	18(23.1)	21(13.5)	*4.97(2.16,11.45)* **** ^**∗∗∗**^	2.94(0.67,12.80)
Good	15(19.2)	87(55.8)	1	1

^*∗*^ *p* < 0.05, ^*∗∗*^*p* < 0.01, and ^*∗∗∗*^*p* < 0.001.

## Data Availability

All data generated or analyzed during this study are included in published article. The data set during the current study is available from the corresponding author upon reasonable request.

## Abbreviations

AOR: Adjusted odd ratio
CI: Confidence interval
CMD: Common mental disorder
LAMIC: Low- and middle-income countries
SRQ: Self-Reporting Questionnaire
WHO: World Health Organization.

## References

[1] The State of the World’s Children A unicef report:childhood under threat.

[2] Caulfield L. E., de Onis M., Blössner M., Black R. E. (2004). Undernutrition as an underlying cause of child deaths associated with diarrhea, pneumonia, malaria, and measles. *American Journal of Clinical Nutrition*.

[3] World Bank: repositioning nutrition as central to development: a strategy for large-scale action. http://siteresources.worldbank.org/NUTRITION/Resources/281846-1131636806329/NutritionStrategy.pdf%5d&gt.

[4] Black R. E., Allen L. H., Bhutta Z. A. (2008). Maternal and child undernutrition: global and regional exposures and health consequences. *The Lancet*.

[5] UNICEF (2008). *The State of the World's Children 2009: Maternal And Newborn Health*.

[6] Engle P. L., Menon P., Garrett J. L., Slack A. (1997). Urbanization and caregiving: a framework for analysis and examples from southern and eastern Africa. *Environment and Urbanization*.

[7] Prince M., Patel V., Saxena S. (2007). No health without mental health. *The Lancet*.

[8] Wachs T. D., Black M. M., Engle P. L. (2009). Maternal depression: a global threat to children's health, development, and behavior and to human rights. *Child Development Perspectives*.

[9] WHO | the world health report 2001 - mental health: new understanding, new hope. http://www.who.int/whr/2001/en/whr01_en.pdf?ua=1&gt.

[10] Lund C. (2010). Mental health in africa: findings from the mental health and poverty project. *International Review of Psychiatry*.

[11] Patel V., Kleinman A. (2003). Poverty and common mental disorders in developing countries. *Bulletin of the World Health Organization*.

[12] Bennett H. A., Einarson A., Taddio A., Koren G., Einarson T. R. (2004). Prevalence of depression during pregnancy: systematic review. *Obstetrics & Gynecology*.

[13] Ross J., Hanlon C., Medhin G. (2011). Perinatal mental distress and infant morbidity in Ethiopia: a cohort study. *ADC - Fetal and Neonatal Edition*.

[14] Cook J. T., Frank D. A., Berkowitz C. (2004). Food insecurity is associated with adverse health outcomes among human infants and toddlers. *Journal of Nutrition*.

[15] Surkan P. J., Kennedy C. E., Hurley K. M., Black M. M. (2011). Maternal depression and early childhood growth in developing countries: systematic review and meta-analysis. *Bulletin of the World Health Organization*.

[16] Rahman A., Surkan P. J., Cayetano C. E., Rwagatare P., Dickson K. E. (2013). Grand challenges: integrating maternal mental health into maternal and child health programmes. *PLoS Medicine*.

[17] Gelaye B., Rondon M. B., Araya R., Williams M. A. (2016). Epidemiology of maternal depression, risk factors, and child outcomes in low-income and middle-income countries. *The Lancet Psychiatry*.

[18] Herba C. M., Glover V., Ramchandani P. G., Rondon M. B. (2016). Maternal depression and mental health in early childhood: an examination of underlying mechanisms in low-income and middle-income countries. *The Lancet Psychiatry*.

[19] Emerson J. (2017). *Maternal Mental Health and Maternal and Child Nutrition in Eastern Democratic Republic of Congo: A Mixed Methods Study [Doctoral dissertation]*.

[20] Patel V., DeSouza N., Rodrigues M. (2003). Postnatal depression and infant growth and development in low income countries: a cohort study from Goa, India. *Archives of Disease in Childhood*.

[21] Minkovitz C. S., Strobino D., Scharfstein D. (2005). Maternal depressive symptoms and children's receipt of health care in the first 3 years of life. *Pediatrics*.

[22] Field T. (1998). Maternal depression effects on infants and early interventions. *Preventive Medicine*.

[23] Patel V., Rahman A., Jacob K. S., Hughes M. (2004). Effect of maternal mental health on infant growth in low income countries: new evidence from South Asia. *British Medical Journal*.

[24] Engle P., Bentley M., Pelto G. (2000). The role of care in nutrition programmes: current research and a research agenda. *Proceedings of the Nutrition Society*.

[25] Frank D. A., Zeisel S. H. (1988). Failure to thrive. *Pediatric Clinics of North America*.

[26] Ruel M. T., Levin C. E., Armar-Klemesu M., Maxwell D., Morris S. S. (1999). Good care practices can mitigate the negative effects of poverty and low maternal schooling on children's nutritional status: evidence from accra. *World Development*.

[27] Harpham T., Huttly S., De Silva M. J., Abramsky T. (2005). Maternal mental health and child nutritional status in four developing countries. *Journal of Epidemiology and Community Health*.

[28] Rahman A., Lovel H., Bunn J., Iqbal Z., Harrington R. (2004). Mothers' mental health and infant growth: a case-control study from rawalpindi, pakistan. *Child: Care, Health and Development*.

[29] Black M. M., Baqui A. H., Zaman K., El Arifeen S., Black R. E. (2009). Maternal depressive symptoms and infant growth in rural Bangladesh. *American Journal of Clinical Nutrition*.

[30] ICF CSA. *Ethiopian demographic and health survey 2016*.

[31] Rauh V. A., Wasserman G. A., Brunelli S. A. (1990). Determinants of maternal child-rearing attitudes. *Journal of the American Academy of Child and Adolescent Psychiatry*.

[32] Anoop S., Saravanan B., Joseph A., Cherian A., Jacob K. S. (2004). Maternal depression and low maternal intelligence as risk factors for malnutrition in children: a community based case-control study from south india. *Archives of Disease in Childhood*.

[33] Melaku N. (2007). *Agro Ecological comparison of Levels and Correlates of Nutritional Status of Under Five Children in Dara Woreda of Sidama Zone, SNNPR [master, thesis]*.

[34] Teshome B., Kogi-Makau W., Getahun Z., Taye G. (2010). Magnitude and determinants of stunting in children underfive years of age in food surplus region of Ethiopia: the case of West Gojam Zone. *Ethiopian Journal of Health Development*.

[35] Cooper P. J., Murray L. (1998). Fortnightly review. Postnatal depression. *British Medical Journal*.

[36] Martins C., Gaffan E. A. (2000). Effects of early maternal depression on patterns of infant-mother attachment: a meta-analytic investigation. *Journal of Child Psychology and Psychiatry and Allied Disciplines*.

[37] Rahman A., Harrington R., Bunn J. (2002). Can maternal depression increase infant risk of illness and growth impairment in developing countries?. *Child: Care, Health and Development*.

[38] Rahman A., Iqbal Z., Bunn J., Lovel H., Harrington R. (2004). Impact of maternal depression on infant nutritional status and illness: a cohort study. *Archives of General Psychiatry*.

[39] Vozoris N. T., Tarasuk V. S. (2003). Household food insufficiency is associated with poorer health. *Journal of Nutrition*.

[40] Huddleston-Casas C., Charnigo R., Simmons L. A. (2009). Food insecurity and maternal depression in rural, low-income families: a longitudinal investigation. *Public Health Nutrition*.

[41] Sathyanarayana Rao T., Asha M. R., Ramesh B. N., Jagannatha Rao K. S. (2008). Understanding nutrition, depression and mental illnesses. *Indian Journal of Psychiatry*.

[42] Beck A., Crain A. L., Solberg L. I. (2011). Severity of depression and magnitude of productivity loss. *Annals of Family Medicine*.

[43] Rahman A., Bunn J., Lovel H., Creed F. (2007). Maternal depression increases infant risk of diarrhoeal illness: - a cohort study. *Archives of Disease in Childhood*.

[44] Guo N., Bindt C., Bonle M. T. (2013). Association of antepartum and postpartum depression in ghanaian and ivorian womenwith febrile illness in their offspring: a prospective birth cohort study. *American Journal of Epidemiology*.

[45] Thongkrajai E., Thongkrajai P., Stoeckel J., Na-nakhon S., Karenjanabutr B., Sirivatanamethanont J. (2016). Socioeconomic and Health Programme Effects Upon the Behavioral Management of Diarrhoeal Disease in Northeast Thailand. *Asia Pacific Journal of Public Health*.

